# Neutrophils Mediate Pulmonary Artery Thrombosis In Situ

**DOI:** 10.3390/ijms23105829

**Published:** 2022-05-23

**Authors:** Olga Porembskaya, Vsevolod Zinserling, Vladimir Tomson, Yana Toropova, Eleonora A. Starikova, Vitaliy V. Maslei, Nika I. Bulavinova, Olga V. Kirik, Marina A. Syrtsova, Leonid Laberko, Maxim I. Galchenko, Vyacheslav Kravchuk, Sergey Saiganov, Alexander Brill

**Affiliations:** 1 Cardio-Vascular Department, Mechnikov North-Western State Medical University, 191015 Saint Petersburg, Russia; olgaporembskaya@gmail.com (O.P.); vyacheslavkravchuk@szgmu.ru (V.K.); sergey.sayganov@szgmu.ru (S.S.); 2 Institute of Experimental Medicine, Almazov National Medical Research Center, 197341 Saint Petersburg, Russia; zinserling@yandex.ru (V.Z.); yana.toropova@mail.ru (Y.T.); vitalijmaslej04@gmail.com (V.V.M.); doctor-niks@mail.ru (N.I.B.); 3 Scientific and Research Center, Pavlov University, 197022 Saint Petersburg, Russia; nic.spb@mail.ru; 4 Department of Immunology, Institute of Experimental Medicine, 197376 Saint Petersburg, Russia; starickova@yandex.ru (E.A.S.); olga_kirik@mail.ru (O.V.K.); 5 Novartis, 194362 Saint Petersburg, Russia; marina.syrczova@mail.ru; 6 Department of General Surgery and Radiology, Pirogov Russian National Research Medical University, 117997 Moscow, Russia; laberko@list.ru; 7 Department of Electrical Engineering and Electrical Equipment, State Agrarian University, 196601 Saint Petersburg, Russia; maxim.galchenko@gmail.com; 8 Institute of Cardiovascular Sciences, College of Medical and Dental Sciences, University of Birmingham, Birmingham B15 2TT, UK

**Keywords:** deep vein thrombosis, pulmonary embolism, neutrophils

## Abstract

Pulmonary embolism is a life-threatening condition, which can result in respiratory insufficiency and death. Blood clots occluding branches of the pulmonary artery (PA) are traditionally considered to originate from thrombi in deep veins (usually in legs). However, growing evidence suggests that occlusion of the vessels in the lungs can develop without preceding deep vein thrombosis (DVT). In this work, we used an inferior vena cava (IVC) complete ligation model of DVT in Wistar rats to explore the possibility and mechanisms of PA thrombosis under the conditions where all routes of thrombotic mass migration from peripheral veins are blocked. We demonstrate that rats both with normal and reduced neutrophil counts developed thrombi in the IVC, although, neutropenia caused a substantial decrease in thrombus size and a shift from fresh fibrin toward mature fibrin and connective tissue inside the thrombus. Massive fibrin deposition was found in the PA branches in the majority of DVT rats with normal neutrophil counts, but in none of the neutropenic animals. Neutrophil ablation also abolished macroscopic signs of lung damage. Altogether, the results demonstrate that thrombi in the lung vasculature can form in situ by mechanisms that require local neutrophil recruitment taking place in the DVT setting.

## 1. Introduction

Pulmonary embolism (PE) is a life-threatening condition with high mortality. The migration of thrombotic masses from deep veins to the pulmonary circulatory bed has long been considered the main mechanism of PE development. However, pulmonary artery (PA) occlusion frequently occurs without initial peripheral thrombosis, which implies that thrombi can form in the PA in situ rather than being delivered as emboli from elsewhere. For example, isolated PA thrombosis has been reported in COPD, surgeries and other conditions [1]. Isolated thrombosis of the PA has clinical and morphological characteristics distinct from PE [2]. We have recently presented a hypothesis that thrombosis can develop in the branches of the PA mechanistically independently of thrombotic mass translocation from deep veins [3]. Pulmonary thrombosis can develop in situ caused by the activation and procoagulant shift of the endothelium, which results from the systemic proinflammatory conditions usually leading to venous thrombosis. The role of sterile inflammation in thrombosis was demonstrated a decade ago [4,5]. In the absence of endothelial denudation, typical for atherosclerotic plaque rupture in the arteries, inflammatory mechanisms including release of the Weibel–Palade body constituents, expression of adhesion receptors, and recruitment of platelets and leukocytes, become critical for thrombosis initiation. This systemic proinflammatory shift can be triggered by cytokines, high molecular group box 1 (HMGB1), neutrophil extracellular traps (NETs), microparticles and other factors [3,6,7,8,9,10]. Different endothelium-associated factors, such as thrombomodulin, protein C, Plasminogen Activator Inhibitor I (PAI-I), von Willebrand factor or contact activation of the coagulation cascade can also play a role [11]. All these factors, separately or in different combinations, can create a systemic proinflammatory milieu promoting isolated fibrin deposition in PA branches.

In this study, we report the principal possibility and neutrophil-dependent mechanisms of local thrombosis in the PA independent of embolism of the peripheral venous thrombus. Our findings carry a clinically important message implying that at least a part of patients at risk could develop life-threatening pulmonary occlusion via mechanisms distinct from the simple transfer of thrombotic masses from the periphery, and that these mechanisms might represent a promising target to reduce morbidity and mortality of the disease.

## 2. Results

### 2.1. Neutropenia Induces Formation of Smaller IVC Thrombi

**Figure 1:**
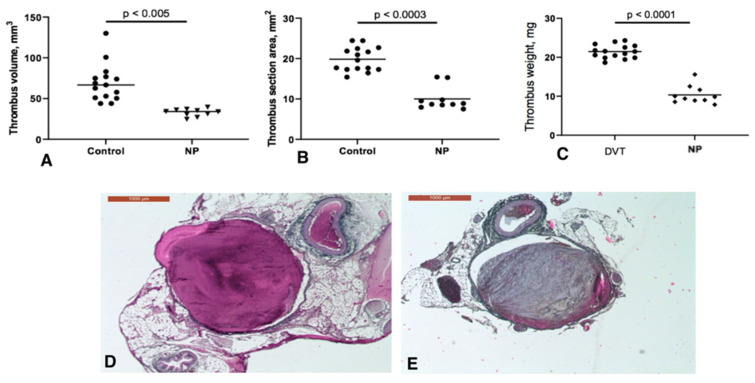
Thrombosis in the IVC: Volume (**A**), area (**B**) and weight (**C**) of thrombi in normal (Control) and neutropenic (NP) conditions. Horizontal line represents median. (**D**,**E**), sections of thrombi in the IVC of non-neutropenic and neutropenic rats, respectively, stained by the Martius Scarlet Blue (MSB) method. Red, fresh fibrin; blue, organized fibrin/connective tissue. Scale bar 1 mm. Representative images out of 10–15 experiments.

To test our hypothesis about in situ pulmonary thrombosis experimentally, we modelled venous thrombosis in Wistar rats by the complete ligation of the inferior vena cava (IVC) for 48 h and evaluated clots in both the PA and the IVC in normal vs. neutropenic conditions created 24 h after surgery. Our results show that all rats both with (*n* = 10) and without (*n* = 15) neutropenia developed thrombi in the IVC. Thrombus area, volume and weight were reduced in neutropenic rats from 19.8 ± 2.9 to 10.5 ± 2.9 mm^2^ (*p* < 0.003), from 69.2 ± 23 to 32.3 ± 4.6 mm^3^ (*p* < 0.005) and from 21.5 ± 1.7 to 10.3 ± 2.3 mg (*p* < 0.0001), respectively (Figure 1A–C). No difference in thrombus size was observed between male and female animals (thrombus weight 21.3 ± 1.8 mg vs. 21.6 ± 1.7 mg in males and females, respectively, *p* = 0.7). No thrombi were observed in visible branches of the IVC in both groups. Sham-operated (*n* = 10) and intact (*n* = 10) animals did not have a thrombus in the IVC.

### 2.2. Neutrophil Depletion Accelerates Fibrin Organization in the IVC Thrombus

Histologically, staining by the Martius Scarlet Blue (MSB) method showed that thrombi in the IVC of non-neutropenic rats consisted predominantly of fresh fibrin with small incorporations of organized fibrin and connective tissue (median 1.2%, interquartile range 0.7–7.3% of thrombus area; Figure 1D). In contrast, thrombi in the animals with neutropenia contained large amounts of organized fibrin and connective tissue (median 46%, interquartile range 38.8–63%, *p* < 0.0001; Figure 1E).

### 2.3. Neutrophil Depletion Prevents Pulmonary Thrombosis

**Figure 2:**
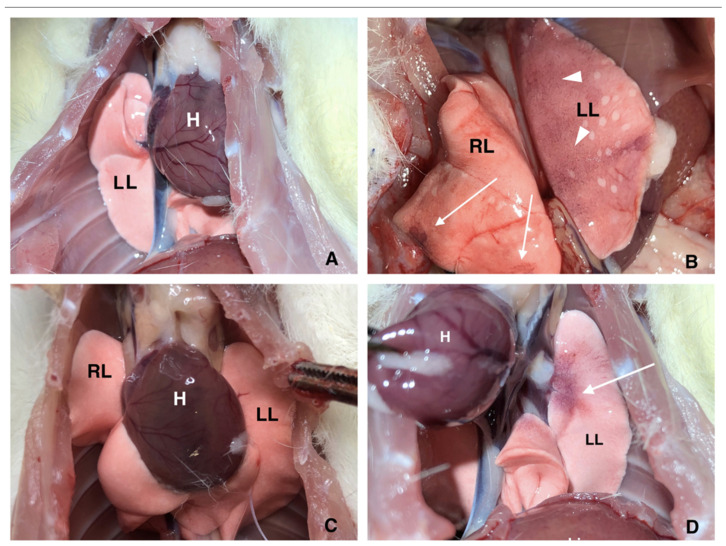
Macroscopic changes in the rat lungs: (**A**), normal intact rat lung without macroscopic changes. (**B**), diffuse (arrowheads) and local (arrows) sites of cyanosis; rat with normal neutrophil count after 48 h IVC ligation. (**C**), no signs of lung injury/malfunction in a neutropenic rat after 48 h IVC stenosis; (**D**), sporadic local site of cyanosis (arrow) near lung root in the sham-operated rat. RL, right lung; LL, left lung; H, heart. Representative images out of 10–15 experiments.

Macroscopic examination of the lungs revealed no signs of ischemia in the unchallenged animals, whose lungs had homogenous color and normal air filling (Figure 2A). In contrast, thrombosis in the IVC was accompanied by massive signs of ischemia throughout both lungs, large areas of ischemia, diffuse cyanosis and reduced air filling of the lung tissue (Figure 2B). All these signs were no longer observed in neutropenic animals, whose lungs looked very similar to the lungs of intact rats (Figure 2C). Interestingly, compared to the lungs of intact rats, sham surgery induced the appearance of sporadic local sites of cyanosis located predominantly near the lung root in a proportion of animals (Figure 2D). Although these changes were by far smaller than those observed in the lungs of rats after IVC ligation, these findings imply that the fact of major surgery is not innocent and sham controls are absolutely necessary in such types of in vivo experiments.

**Figure 3:**
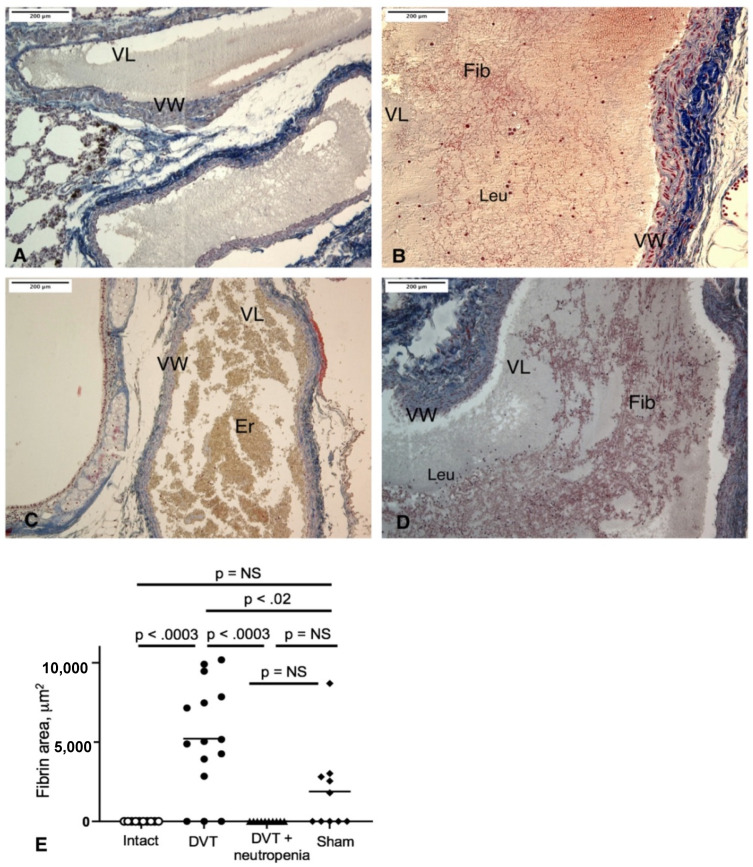
Microscopic changes in the branches of the PA (**A**–**D**) and area of fibrin in PA branches (**E**): (**A**), PA branch in an intact rat without thrombus inside. (**B**), PA branches of a non-neutropenic rat after 48 h IVC ligation, with firmly packed young fibrin meshwork adjacent to the vessel wall and occluding most of the vessel lumen. (**C**), PA branch of a neutropenic rat after 48 h IVC ligation, free of fibrin deposition, with yellow erythrocytes inside the vessel. (**D**), Separate loose young fibrin fibers in the PA branch of a sham-operated rat. (**E**), Area of fibrin in PA branches was measured by ImageJ in random fields of view (one per section). Statistical comparison was performed by Kruskal–Wallis test. NS, non-significant. N = 10 (intact), 15 (48 h IVC stenosis/DVT), 10 (48 h IVC stenosis/DVT + neutropenia), 10 (sham). VW, vessel wall; VL, vessel lumen; Leu, leukocytes; Fib—fibrin, Er—erythrocytes. Scale bar 200 μm. Representative images out of 10–15 experiments.

We next analyzed fibrin deposition in PA branches comparable in size (Supplementary Figure S1). No thrombi or fibrin deposition was detected in the lungs of intact animals (Figure 3A). Thrombi were found in large PA branches of both lungs in 12 out of 15 rats with normal neutrophil counts, whereas none of the neutropenic animals had thrombi in the lungs (*p* < 0.002 by the Fisher’s exact test; fibrin area median 5033 vs. 0 μm^2^, *p* < 0.003; Figure 3B,C). Interestingly, thrombi in the PA were absent specifically in those 3 out of 15 non-neutropenic rats, which had substantially larger amounts of connective tissues in their IVC thrombi. Thrombi in the PA consisted predominantly of a young fibrin network fixed to the vessel wall and occluding most of the vessel lumen. Polymorphonuclear leukocytes were either dissipated in the vessel lumen or accumulated near the endothelium, which did not show any signs of denudation. In the lungs of sham-operated animals, no solid thrombi were observed, although in some of the rats, a small number of young fibrin fibers was discovered suggesting that a major surgery alone can provoke a certain degree of systemic procoagulant conditions (Figure 3D,E; fibrin area median 5033 vs. 901 μm^2^, *p* < 0.02 in DVT vs. sham-operated rats, respectively).

## 3. Discussion

Our study demonstrates that blood clots can occlude even large branches of the PA in the conditions, in which direct migration of the whole thrombus or its parts from the IVC to the lungs is excluded. Indeed, full ligation completely blocks blood flow in the IVC, which precludes movement of fibrin fibrils pre-formed in the IVC to the pulmonary circulation [12,13]. It is known, however, that a system of collaterals exists in both humans and rodents, including gonadal, superficial and internal epigastric and periureteric veins [14]. The involvement of these collateral pathways in spreading thrombus material cannot be completely ruled out; although, it is unlikely given the high proportion and large size of the occluded PA branches. Moreover, in this study, we have tested thrombosis specifically in the lungs because of its high clinical importance and apparent proven epidemiological association with deep vein thrombosis (DVT). This does not exclude the possibility that thrombi could also develop in other organs and vascular beds.

A frequently utilized animal model of PE is the infusion of platelet-activating substances, such as a collagen–epinephrine mixture, histones etc., after which wild-type animals rapidly (within minutes) die of respiratory insufficiency, and massive thrombosis is observed thereafter in the lung sections [10,15,16]. However, it is important to note that in this approach, lung thrombosis largely reflects platelet activation and is virtually entirely platelet-dependent and therefore cannot adequately recapitulate PE in humans. In contrast, our observation of pulmonary clots in the setting of blood flow restriction in a large vein mimics a real clinical situation, in which venous thromboembolism can result from prolonged immobilization, which leads to blood flow stagnancy. Better congruity of our model is further supported by the structure of the thrombi consisting predominantly of fibrin than aggregated platelets, which is typical for PE clots [2].

In humans, thrombi in the PA and veins are structurally different. For example, PA clots contain larger quantities of fibrin, leukocytes and microparticles compared to venous thrombi [2]. This suggests that both systemic and local conditions, such as vascular endothelial cell dysfunction, hypoxia, and inflammation, likely provoke thrombosis directly in the pulmonary circulation [17]. Such systemic conditions can be mediated by circulating blood cells. For example, platelets playing an important role in venous thrombosis, can upon activation stimulate the release of Weibel–Palade bodies from endothelial cells thus increasing their proinflammatory and prothrombotic potential [18]. The role of platelet activation in pulmonary circulation obstruction was demonstrated in experiments, in which mice deficient in thrombin receptors PAR3 or PAR4 were protected from respiratory insufficiency and death [19]. Platelets can release HMGB1, which activates endothelial cells inducing expression of adhesion receptors and secretion of proinflammatory substances [9]. Under the influence of HMGB1, aggregates can form and occlude branches of the PA [7]. In addition, platelet-derived HMGB1 stimulates the release of NETs, which promote activation of the endothelium and coagulation cascade, mediate lung injury and facilitate thrombosis [20].

We demonstrate here that neutrophil ablation strongly reduces thrombus size in the IVC and protects mice from thrombi in the PA. This corroborates previous reports demonstrating the inhibitory effect on venous thrombosis of both NETs destruction by DNAse and prevention of their formation (e.g., by inhibition of peptidylarginine deiminase 4) [20]. NETs promote thrombosis in a variety of cardiovascular diseases through such mechanisms as stimulation of tissue factor expression, thrombus stabilization, activation of the endothelium and platelets, and others [21]. NETs are present in PA thrombi both in COVID-19 and in the absence of infection [22,23]. Increased plasma levels of histones, an integral component of NETs, lead to thrombotic obstruction of PA branches in trauma [24]. NETs also enhance fibrotic thrombus remodeling through a TGF-β-dependent mechanism, thus preventing thrombosis resolution [25]. Neutrophils can synthesize and release multiple substances able to activate endothelium and support the coagulation cascade. For example, massive deposition of fibrin around adherent neutrophils under venous shear has been reported [26]. This effect was dependent on Factor XII, elastase and cathepsin G, and dramatically increased in the presence of platelets suggesting that neutrophils could drive clotting in the lungs in our experiments. Neutrophil α-defensins support fibrin formation and venous thrombosis in mice [27]. Fibrin, in turn, stimulates major functions of neutrophils, such as the production of ROS and NETosis, thus forming a fibrin/neutrophil positive feedback loop that can further promote thrombosis [28]. Moreover, NETs and NLRP3 inflammasome can form a “vicious circle” supporting DVT [29]. Neutrophil elastase (which is a part of NETs) is able to activate the coagulation Factor XIII rendering it less susceptible to proteolytically inactivation [30]. FXIII, in turn, mediates retention of red blood cells in venous thrombi thus supporting thrombosis, whereas deficiency in FXIII increases the incidence of PE in the IVC ligation/de-ligation model [31,32,33]. Fluctuations of FXIII levels accompany thrombotic accidents implying both its activation and consumption during the event [34]. The absence of all these mechanisms in the setting of neutrophil depletion could result in the reduction in thrombus mass in the IVC and abolishment of fibrin deposition in the PA.

Interestingly, it has been reported that neutropenia increases thrombus size in the rat IVC on days 2 and 7 after stasis application^4^. This discrepancy could be attributed to different regimens of neutrophil depletion (prior to surgery in [35] vs. 24 h post-surgery in our experiments), although, clarification of precise mechanisms of this apparent ambiguous neutrophil impact on venous thrombosis requires further study. In a partial IVC ligation model in mice, neutrophil depletion suppressed thrombosis [5], which corroborates our results. As neutrophils are critical for venous thrombus development, we induced neutropenia 24 h after induction of blood stasis to allow IVC thrombi to form. This experimental design was chosen to rule out the absence of initial thrombi in the vein as a potential explanation for reduced thrombosis in the PA. Neutrophil depletion resulted in the accumulation of organized fibrin and fibrosis in the IVC thrombi. This may be explained, at least partially, by reduced fibrinolytic activity normally associated with neutrophils (such as uPA secretion and degradation of PAI-1 by neutrophil elastase) and decreased collagenase potential mediated by neutrophil metalloproteinases and elastase [36]. Fluctuating levels of FXIII can modify the effect of collagenase on the survival of metabolically active fibroblasts, cells predominantly responsible for the fibrotic shift, and this mechanism could also potentially be involved [37].

In conclusion, we have demonstrated herein a fundamental possibility of local thrombosis in the PA, driven by neutrophil accrual accompanying thrombus formation in the vein. Neutrophils are key players in this process and can therefore be considered potential targets for the prevention of pulmonary vascular bed obstruction in patients at high risk for thrombosis.

## 4. Materials and Methods

### 4.1. DVT Surgery

All animal experiments were approved by the ethical committee of the North-Western State Medical University, St. Petersburg, Russia (protocol No. 7 of 7 October 2020). Thrombosis was induced in Wistar rats of both sexes (around 300 g body weight) by complete ligation of the IVC and all visible branches as described [35]. In brief, rats were anesthetized by oxygen–isoflurane mixture, median laparotomy was performed, and intestines exteriorized. IVC was gently separated from aorta and completely ligated with a polypropylene suture immediately below the left renal vein. All side and back branches were completely closed as well. Then, the abdominal cavity was washed with an antiseptic solution and the peritoneum and skin were closed with a suture. None of the animals died as a result of surgery. All rats remained active and did not demonstrate signs of discomfort, and the time of surgery was comparable in all experiments. Rats were humanely culled after 48 h and thrombi were excised for further analysis. Thrombus weight was measured after dehydration procedures preceding paraffin embedding to obtain weight of the dry tissue.

### 4.2. Induction of Neutropenia

Neutrophil depletion was induced by i.p. injection of an anti-neutrophil antibody (antiPMN; AIAD51140; Accurate Chemical Co., Westbury, NY, USA; 0.2 mL/100 g body weight) as described before [38] with some modifications. Dramatic reduction of the neutrophil count was confirmed by FACS in all rats of the group (Supplementary Figure S2).

### 4.3. Histology

For evaluation of the structure of IVC thrombi, sections from the proximal third of the thrombus were utilized. Sections taken from both lungs in the areas close to the lung root were used for assessment of the pulmonary vascular bed. Staining of paraffin-embedded sections was performed by the Martius Scarlet Blue (MSB) method, in which young fibrin has a pink color, whereas organized fibrin and collagen fibers are blue; RBCs are yellow. Sections were photographed by a microscope Leica HC with camera Leica DMC 2900. For assessment of the area of fibrin in PA branches, ROIs were manually generated and measured using ImageJ (National Institute of Health, Bethesda, MD, USA) [39] in random fields of view (one per section). Statistical comparison was performed by Kruskal–Wallis test.

### 4.4. Statistics

Data were analyzed by IBM SPSS Statistics and GraphPad Prism version 9.3.1. Comparison between groups was performed using unpaired Student’s *t*-test, Mann–Whitney test, or Kruskal–Wallis test. *p* values < 0.05 were considered significant.

## Data Availability

Available upon reasonable request.

## Supplementary Materials

The following supporting information can be downloaded at: https://www.mdpi.com/article/10.3390/ijms23105829/s1.
