# Supplementary material for: Neutrophils Mediate Pulmonary Artery Thrombosis In Situ
Source: Int J Mol Sci. 2022 May 23;23(10):5829. doi: 10.3390/ijms23105829 (PMC9144243; doi:10.3390/ijms23105829)
Supplement: Supplementary file 1 [file ijms-23-05829-s001.zip › ijms-1707148-supplementary.pdf]

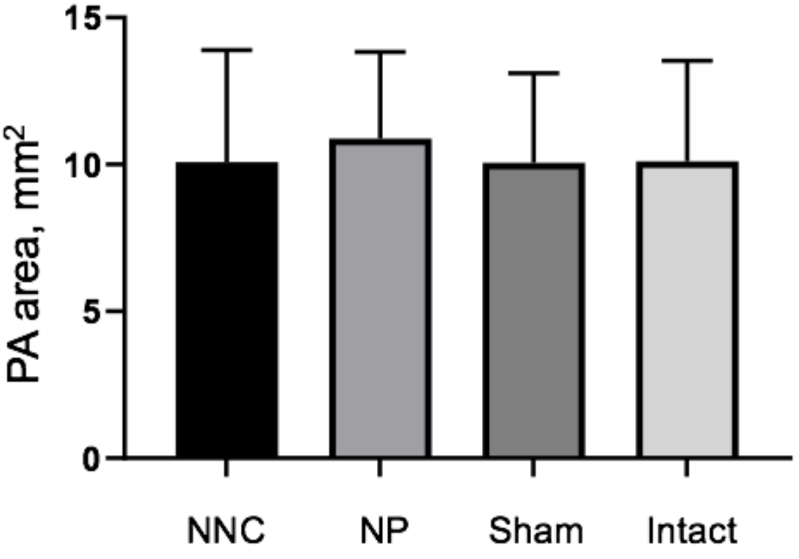

Supplementary Figure S1. Area of the analyzed branches of the PA.
NNC, normal neutrophil counts; NP, neutropenia. Mean ± STD; no significant differences between any of the groups were observed; n = 10-15.

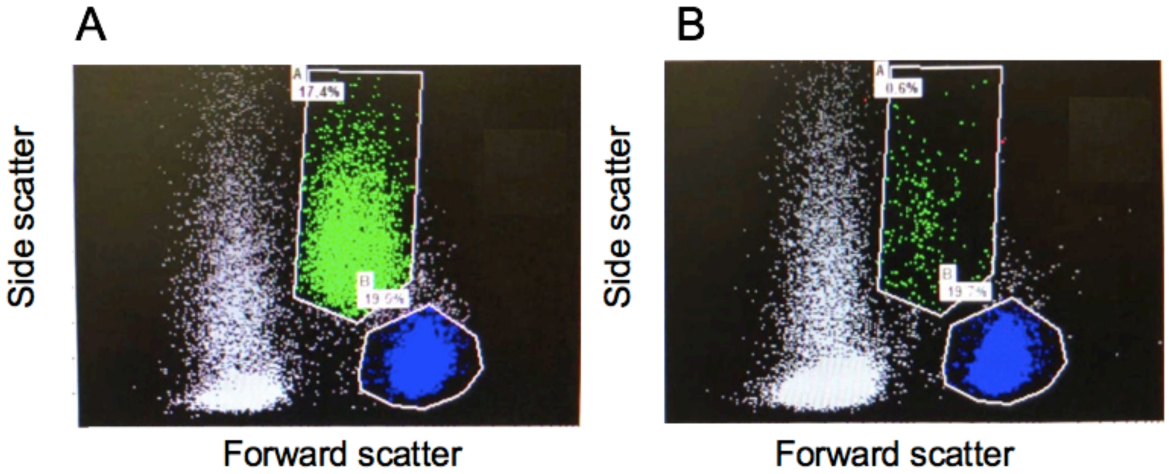

Supplementary Figure S2. Neutrophil depletion confirmed by FACS.
Green gated events represent neutrophils without (A) or after (B) anti-neutrophil antibody administration. Other leukocyte counts remained unchanged (white, lymphocytes; blue, monocytes). A representative image is shown.
